# Histopathological tumour response scoring in resected pancreatic cancer following neoadjuvant therapy: international interobserver study (ISGPP-1)

**DOI:** 10.1093/bjs/znac350

**Published:** 2022-11-04

**Authors:** Boris V Janssen, Stijn van Roessel, Susan van Dieren, Onno de Boer, Volkan Adsay, Olca Basturk, Lodewijk Brosens, Fiona Campbell, Deyali Chatterjee, Angela Chou, Claudio Doglioni, Irene Esposito, Roger Feakins, Talia L Fuchs, Noriyoshi Fukushima, Anthony J Gill, Seung-Mo Hong, Ralph H Hruban, Jeffrey Kaplan, Alyssa Krasinkas, Claudio Luchini, Chanjuan Shi, Aatur Singhi, Elizabeth Thompson, Marie-Louise F Velthuysen, Marc G Besselink, Joanne Verheij, Huamin Wang, Caroline Verbeke, Arantza Fariña, for the International Study Group of Pancreatic Pathologists (ISGPP)

**Affiliations:** Department of Surgery, Amsterdam UMC, location University of Amsterdam, Amsterdam, the Netherlands; Department of Pathology, Amsterdam UMC, location University of Amsterdam, Amsterdam, the Netherlands; Cancer Centre Amsterdam, Amsterdam, the Netherlands; Department of Surgery, Amsterdam UMC, location University of Amsterdam, Amsterdam, the Netherlands; Cancer Centre Amsterdam, Amsterdam, the Netherlands; Department of Surgery, Amsterdam UMC, location University of Amsterdam, Amsterdam, the Netherlands; Cancer Centre Amsterdam, Amsterdam, the Netherlands; Department of Pathology, Amsterdam UMC, location University of Amsterdam, Amsterdam, the Netherlands; Cancer Centre Amsterdam, Amsterdam, the Netherlands; Department of Pathology, Koc University and KUTTAM Research Centre, Istanbul, Turkey; Department of Pathology, Memorial Sloan Kettering Cancer Center, New York, New York, USA; Department of Pathology, University Medical Centre Utrecht, Utrecht, the Netherlands; Department of Pathology, Royal Liverpool University Hospital, Liverpool, UK; Department of Anatomical Pathology, University of Texas MD Anderson Cancer Center, Houston, Texas, USA; Cancer Diagnosis and Pathology Group, Kolling Institute of Medical Research, Royal North Shore Hospital, St Leonards, and University of Sydney, Sydney, New South WalesAustralia; Department of Pathology, IRCCS San Raffaele Scientific Institute, Milan, Italy; Institute of Pathology, Heinrich-Heine-University and University Hospital of Duesseldorf, Duesseldorf, Germany; Department of Pathology, Royal Free London NHS Trust, London, UK; Cancer Diagnosis and Pathology Group, Kolling Institute of Medical Research, Royal North Shore Hospital, St Leonards, and University of Sydney, Sydney, New South WalesAustralia; Department of Pathology, Jichi Medical University Hospital, Tochigi, Japan; Cancer Diagnosis and Pathology Group, Kolling Institute of Medical Research, Royal North Shore Hospital, St Leonards, and University of Sydney, Sydney, New South WalesAustralia; Department of Pathology, Asan Medical Centre, Seoul, Korea; Department of Pathology, Sol Goldman Pancreatic Cancer Research Center, Johns Hopkins University School of Medicine, Baltimore, Maryland, USA; Department of Pathology, University of Colorado Hospital, Denver, Colorado, USA; Department of Pathology, Emory University, Atlanta, Georgia, USA; Department of Diagnostics and Public Health, University and Hospital Trust of Verona, Verona, Italy; Department of Pathology, Duke University Medical Center, Durham, North Carolina, USA; Department of Pathology, University of Pittsburgh School of Medicine, Pittsburgh, Pennsylvania, USA; Department of Pathology, Sol Goldman Pancreatic Cancer Research Center, Johns Hopkins University School of Medicine, Baltimore, Maryland, USA; Department of Pathology, Erasmus Medical Centre, Rotterdam, the Netherlands; Department of Surgery, Amsterdam UMC, location University of Amsterdam, Amsterdam, the Netherlands; Cancer Centre Amsterdam, Amsterdam, the Netherlands; Department of Pathology, Amsterdam UMC, location University of Amsterdam, Amsterdam, the Netherlands; Cancer Centre Amsterdam, Amsterdam, the Netherlands; Department of Anatomical Pathology, University of Texas MD Anderson Cancer Center, Houston, Texas, USA; Department of Pathology, Institute of Clinical Medicine, University of Oslo, Oslo, Norway; Department of Pathology, Oslo University Hospital, Oslo, Norway; Department of Pathology, Amsterdam UMC, location University of Amsterdam, Amsterdam, the Netherlands; Cancer Centre Amsterdam, Amsterdam, the Netherlands

## Abstract

**Background:**

Most tumour response scoring systems for resected pancreatic cancer after neoadjuvant therapy score tumour regression. However, whether treatment-induced changes, including tumour regression, can be identified reliably on haematoxylin and eosin-stained slides remains unclear. Moreover, no large study of the interobserver agreement of current tumour response scoring systems for pancreatic cancer exists. This study aimed to investigate whether gastrointestinal/pancreatic pathologists can reliably identify treatment effect on tumour by histology, and to determine the interobserver agreement for current tumour response scoring systems.

**Methods:**

Overall, 23 gastrointestinal/pancreatic pathologists reviewed digital haematoxylin and eosin-stained slides of pancreatic cancer or treated tumour bed. The accuracy in identifying the treatment effect was investigated in 60 patients (30 treatment-naive, 30 after neoadjuvant therapy (NAT)). The interobserver agreement for the College of American Pathologists (CAP) and MD Anderson Cancer Center (MDACC) tumour response scoring systems was assessed in 50 patients using intraclass correlation coefficients (ICCs). An ICC value below 0.50 indicated poor reliability, 0.50 or more and less than 0.75 indicated moderate reliability, 0.75 or more and below 0.90 indicated good reliability, and above 0.90 indicated excellent reliability.

**Results:**

The sensitivity and specificity for identifying NAT effect were 76.2 and 49.0 per cent respectively. After NAT in 50 patients, ICC values for both tumour response scoring systems were moderate: 0.66 for CAP and 0.71 for MDACC.

**Conclusion:**

Identification of the effect of NAT in resected pancreatic cancer proved unreliable, and interobserver agreement for the current tumour response scoring systems was suboptimal. These findings support the recently published International Study Group of Pancreatic Pathologists recommendations to score residual tumour burden rather than tumour regression after NAT.

## Teaser

The ISGPP-1 study demonstrated that identifying the effect of neoadjuvant therapy in resected pancreatic cancer proved unreliable. The interobserver agreement for the current tumour response scoring (TRS) systems was suboptimal. A collaborative effort is required to develop an objective TRS system.

## Introduction

Neoadjuvant therapy (NAT) has been reported to improve disease-free and overall survival^1–5^. Therefore, it is increasingly being used to treat pancreatic ductal adenocarcinoma (PDAC), either primary resectable, borderline resectable, or locally advanced disease. Pancreatic resection specimens are routinely assessed microscopically to determine the effect of NAT. This histological assessment using tumour response scoring (TRS) systems serves a dual purpose. First, as the histological tumour response reflects the sensitivity or resistance of the resected cancer to the NAT, a reliable histological tumour response score may guide the selection of an adjuvant regimen. Second, in the context of RCTs, histological tumour response may act as an objective measure to compare the effectiveness of different NAT regimens^6^.

In past decades, multiple TRS systems for PDAC have been proposed^7^. The TRS systems recommended by the College of American Pathologists (CAP) and the MD Anderson Cancer Center (MDACC) seem to be the most commonly used and studied^7^. The CAP system is a four-tiered descriptive system based on residual cancer relative to tumour regression after NAT. The MDACC system is a three-tiered system based on the percentage of residual viable tumour cells in relation to the treated tumour bed. Both systems identify the areas where the tumour once was and the number of viable tumour cells present (*Table 1*).

**Table 1:** College of American Pathologists and MD Anderson Cancer Center tumour response scoring systems for pancreatic cancer resections after neoadjuvant therapy

Scoring system	Grade	Criteria
College of American Pathologists	0	No viable cancer cells
Washington *et al.*^8,9^	1	Single cells or rare small groups of cancer cells
	2	Residual cancer with evident tumour regression, but more than single cells or rare small groups of cancer cells
	3	Extensive residual cancer with no evident tumour regression
MD Anderson Cancer Center	0	No residual carcinoma
Chatterjee *et al.*^10,11^	1	Minimal residual carcinoma (single cells or small groups of cancer cells; < 5% residual carcinoma in treated tumour bed)
	2	≥ 5% carcinoma in treated tumour bed

In 2020, the International Study Group of Pancreatic Pathologists (ISGPP) issued a consensus statement regarding TRS that may guide the future development of a new TRS system^6^. The ISGPP stated that TRS for surgically resected PDAC after NAT should assess the residual (viable) tumour burden instead of the extent of tumour regression, and the defining criteria of the categories in a TRS system should be objective. However, a large, well controlled study of the interobserver agreement for TRS systems for pancreatic cancer was lacking.

The ISGPP-1 study, therefore, aimed to address these issues, as they are important determinants of the diagnostic value of the current TRS systems. First, the ability of 18 gastrointestinal/pancreatic pathologists to identify NAT effects in routine haematoxylin and eosin-stained slides was tested. Second, interobserver agreement was evaluated for TRS based on the CAP and MDACC TRS systems among 23 gastrointestinal/pancreatic pathologists.

## Methods

### Participating pathologists

Overall, 23 gastrointestinal/pancreatic pathologists of the ISGPP from 9 countries participated. All pathologists who had participated in the Amsterdam International Consensus Meeting on TRS in the pathological assessment of resected pancreatic cancer after NAT were invited to participate, and were also asked to suggest additional pathologists with similar expertise for participation in the present study^6^. The pathologists who attended the consensus meeting were invited based on their expertise in the pathological assessment and TRS of resected pancreatic cancer after NAT.

### Study cohort selection

This study was approved by the institutional review boards of the University of Texas, MDACC (Houston, TX, USA), Oslo University Hospital (Oslo, Norway), and Amsterdam UMC (Amsterdam, the Netherlands). For each part of the study, a separate cohort was selected. Analysis in both cohorts consisted of evaluation of digitalized haematoxylin and eosin-stained slides from patients with PDAC (3 randomly selected tumour slides or tumour bed slides from each patient), aged 18 years or older, who had undergone pancreatoduodenectomy, total pancreatectomy, or distal pancreatectomy for histologically confirmed PDAC. Some patients had not completed the full course of planned NAT before surgery. These patients were not excluded from the study, reflecting actual routine clinical practice.

### Identification of neoadjuvant treatment effect (part 1)

Overall, 60 patients with resected PDAC (30 treatment-naive and 30 after NAT) were included. The patients were identified retrospectively at the Pathology Departments of University of Texas, MDACC (Houston, TX, USA), Oslo University Hospital (Oslo, Norway), and Amsterdam UMC (Amsterdam, the Netherlands). Each centre provided data (patient age, sex, treatment status, treatment type, type of surgery) and 3 haematoxylin and eosin-stained slides for 20 patients with PDAC (10 treated, 10 untreated).

Slide selection was undertaken as follows. First, slides from the macroscopically identified tumour or the tumour bed were included; slides representative of tissues outside the tumour bed were excluded. Also excluded were slides showing evidence of an extended surgical procedure (for example by including part of the superior mesenteric artery or coeliac trunk) and implying that the patient had probably received NAT.

Second, three slides were randomly selected from haematoxylin and eosin-stained slides of the preselected tumour or tumour bed using the True Random Number Generator from random.org^12^. All haematoxylin and eosin-stained slides were collected physically in Amsterdam, scanned using a Philips Intellisite Ultra-Fast Scanner (Philips^®^, Best, the Netherlands), and uploaded to the web-based digital slide platform PathXL Tutor (Cirdan^™^, Lisburn, UK).

All participating pathologists were blinded to treatment status and distribution of treatment-naive patients *versus* those who underwent NAT.

#### Data collection and analysis

Using the online questionnaire functionality of PathXL Tutor, the participating pathologists assessed the 3 slides from all 60 patients and categorized each slide as either treatment-naive or status post-NAT. Each slide was presented individually, in random order rather than grouped per patient. Matrices were created by cross-tabulating treatment status and pathologist assessment. The confusion matrices’ data were pooled to calculate sensitivity, specificity, positive predictive value, and negative predictive value.

The participating pathologists were also asked to grade each slide in terms of level of certainty and difficulty in determining the treatment status. For this purpose, a binary choice option was provided: easy/certain *versus* difficult/uncertain. The proportion of correct predictions per category was calculated.

A final open question was posed: ‘Overall, which histological features helped you make the distinction between treated and treatment-naive cases?’. Answers to the open question were pooled in a catalogue of histological features used to distinguish between treated and treatment-naive cases.

### Interobserver agreement on degree of tumour response (part 2)

Slides from 50 consecutive patients with PDAC who had received NAT and undergone surgical resection at Amsterdam UMC (Amsterdam, the Netherlands) were collected. These patients did not overlap with the previous study set. For each patients, three slides from the tumour bed were randomly selected using the True Random Number Generator^12^. The number of patients and slides were determined in consultation with a statistician based on a minimum sample size calculation for an intraclass correlation (ICC) analysis of 22 patients (performed in R, using the ICC.Sample.Size package^13^). Also taken into account were the distribution between categories (that is over-representation of CAP 2/3 and MDACC 2, potentially introducing bias); and the overall practical feasibility of this project in terms of data storage and time required from the participating pathologists. The selected slides were digitized and uploaded to PathXL. For this part of the study, three slides from a single patient were presented together. Finally, a regimen-based sensitivity analysis was performed by grouping patients with similar neoadjuvant regimens.

#### Data collection and analysis

Using a PathXL digital survey, participating pathologists were asked to score the tumour response to NAT based on the overall evaluation of all three slides from patients treated with NAT using both the CAP and MDACC systems. Patient information, including age, sex, NAT regimen, number of treatment cycles, and surgery type, was collected from medical records. The average interobserver agreement was assessed using ICC analysis. The latter was a two-way analysis (same raters across subjects), measured absolute agreement in terms of average measures, and was based on a random-effects model^14^. The analyses were performed in R using the irr package^15^. An ICC value below 0.50 indicated poor reliability, 0.50 or more and less than 0.75 indicated moderate reliability, 0.75 or more and below 0.90 indicated good reliability, and above 0.90 indicated excellent reliability^16^. Stacked bar charts were generated to illustrate the discrepancies among the participating pathologists for each patient.

## Results

### Study population

Baseline characteristics for both study cohorts are listed in *Tables 2* and *3*. Of 60 patients in part 1, 30 were treated with NAT, and 30 were treatment-naive. In the treated group, the mean age was 64.0 years, 53.3 per cent were women, and 80 per cent underwent pancreatoduodenectomy. In the treatment-naive group, the mean age was 69.6 years, 36.7 per cent were women, and 80 per cent underwent pancreatoduodenectomy. Patients in the NAT group were treated with FOLFIRINOX (combination treatment consisting of leucovorin, fluorouracil, irinotecan, and oxaliplatin) (46.7 per cent), gemcitabine (46.7 per cent), or capecitabine (6.7 per cent). Of these, 36.7 per cent had also received radiotherapy.

**Table 2:** Baseline demographic and clinical characteristics of treatment effect cohort (part 1)

	Untreated patients	Treated patients
	(*n* = 30)	(*n* = 30)
Age at surgery (years), mean (range)	69.6 (44–83)	64.0 (42–77)
Sex ratio (M : F)	19 : 11	14 : 16
**Neoadjuvant therapy**		
Chemotherapy		
FOLFIRINOX ≥ 4 cycles	–	11
FOLFIRINOX < 4 cycles*	**–**	2
Gemcitabine + other ≥ 4 cycles	**–**	5
Gemcitabine + other < 4 cycles	–	1
Chemoradiotherapy		
Gemcitabine ≥ 2 cycles and RTx × 1†	–	7
Gemcitabine < 2 cycles and RTx × 1	–	1
FOLFIRINOX ≥ 4 cycles and RTx × 1	–	1
Capecitabine ≥ 2 cycles RTx × 1	–	1
Capecitabine < 2 cycles RTx × 1	–	1
**Type of surgery**		
Pancreatoduodenectomy	24	24
Distal pancreatectomy	6	4
Total pancreatectomy	0	2

* One patient also received one cycle of FLOX (combination treatment consisting of fluorouracil, leucovorin, and oxaliplatin). †One patient received gemcitabine in combination with oxaliplatin. FOLFIRINOX, combination treatment consisting of leucovorin, fluorouracil, irinotecan, and oxaliplatin; RTx, radiotherapy.

**Table 3:** Baseline demographic and clinical characteristics of interobserver agreement cohort (part 2)

	No. of patients*(*n* = 50)
Age at surgery (years), mean (range)	66.4 (40–83)
Sex ratio (M : F)	26 : 24
**Neoadjuvant therapy**	
Gemcitabine × 3 and RTx × 1	15
FOLFIRINOX ≥ 4 cycles	30
FOLFIRINOX < 4 cycles	3
Gemcitabine/nabpaclitaxel ≥ 3 cycles	2
**Type of surgery**	
Pancreatoduodenectomy	39
Distal pancreatectomy	10
Total pancreatectomy	1

* Unless otherwise indicated. FOLFIRINOX, combination treatment consisting of leucovorin, fluorouracil, irinotecan, and oxaliplatin. RTx, radiotherapy.

Fifty patients treated with NAT were included in part 2 of the study. The mean age was 66.4 years, and 48 per cent were female. Thirty per cent of patients had received gemcitabine-based radiochemotherapy, 66 per cent FOLFIRINOX, and 4 per cent gemcitabine/nab-paclitaxel. The majority had undergone pancreatoduodenectomy (78 per cent).

### Identification of neoadjuvant treatment effect (part 1)

Overall, 18 pathologists from 8 countries participated. Two of 180 slides were excluded because the digital analysis platform erroneously presented them as blacked-out images. Overall, the 18 pathologists made a total of 3204 assessments.

**Fig. 1:**
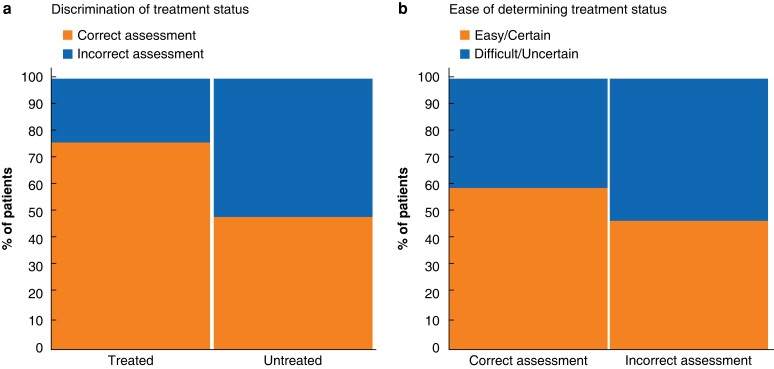
Ability of pathologists to determine treatment status **a** Mean discriminative ability of 18 pathologists to determine treatment status. The mean sensitivity, which is reflected by the percentage of true-positive assessments in the cohort of treated patients, was 76.2 per cent. The mean specificity, which is reflected by the percentage of true-negative assessments in the cohort of untreated patients, was 49.0 per cent. **b** Proportion of correctly and incorrectly scored patients for whom assessment was labelled as ‘easy/certain’ and ‘difficult/uncertain’. Some 59.8 per cent of correctly scored assessments, and 47.7 per cent of incorrectly scored ones, were labelled as ‘easy/certain’.

The mean sensitivity in determining whether a slide was from a patient treated with NAT, and therefore in correctly identifying treatment effect, was 76.2 (range 56.8–97.7) per cent). The mean specificity in determining whether a slide was from a treatment-naive patient and, therefore, correctly identifying a treatment-naive patient as untreated was 49.0 (21.1–66.7) per cent. The mean positive predictive value was 59.4 (53.8–70.4) per cent, and the mean negative predictive value was 67.9 (58.8–92.3) per cent. *Figure 1a* shows the mean sensitivity and specificity, and *Table S1* provides these data for individual pathologists.

**Fig. 2:**
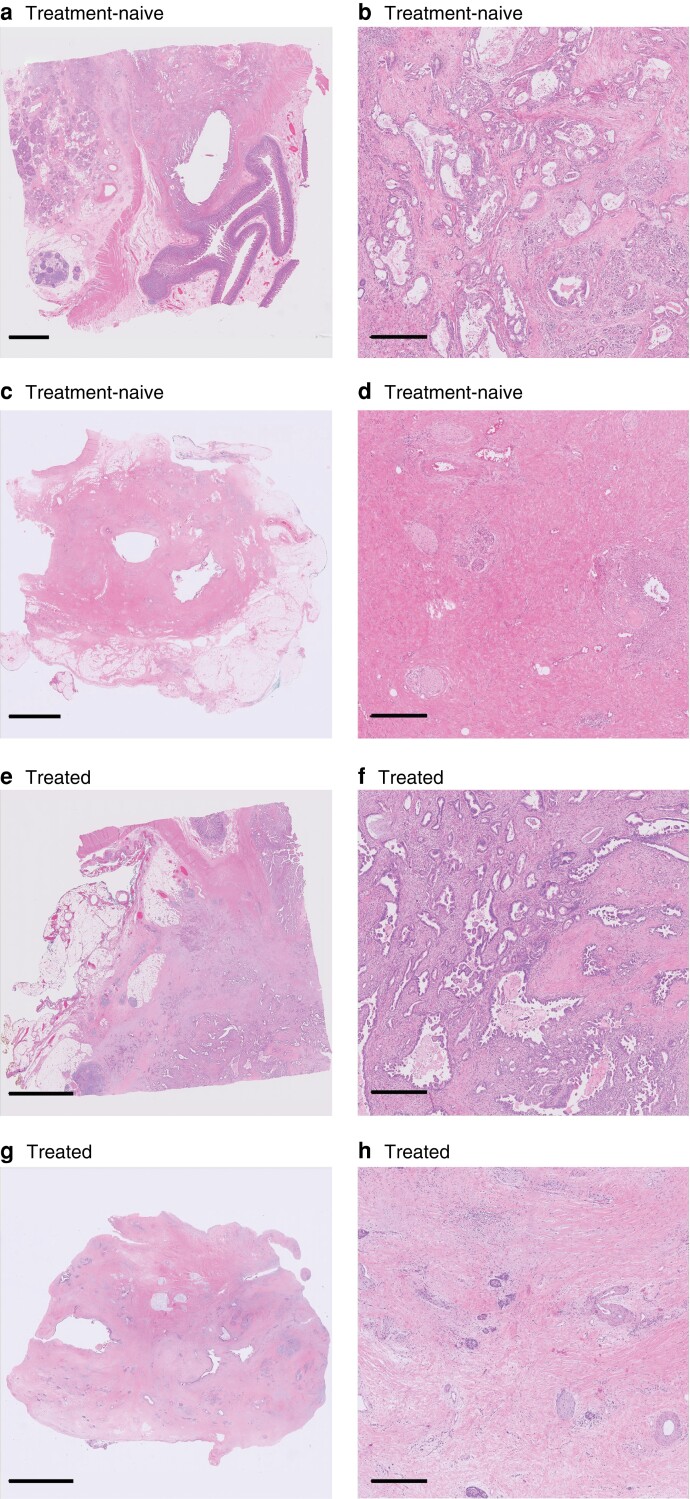
Overlap of histomorphological features between treatment-naive and treated patients **a**,**c** Whole-slide images (WSIs) from treatment-naive patients; **b**,**d** close-up images of sections in these WSIs. **a**,**b** Most pathologists (16 of 18) correctly scored this patient as treatment-naive. **c**,**d** Only 2 of 18 pathologists correctly scored this patient as treatment-naive. **e**,**g** WSIs from treated patients; **f**,**h** close-up images of sections in these WSIs. **e**,**f** Only 5 of 18 pathologists correctly scored this patient as treated. **g**,**h** All pathologists (18 of 18) correctly scored this patient as treated. Scale bars 5 mm (**a**,**c**,**e**,**g**) and 0.5 mm (**b**,**d**,**f**,**h**). All WSIs were scanned at 40× magnification. Sections were stained with haemotoxylin and eosin.

Pathologists considered 55.3 per cent of their assessments as easy/certain. Of these, the treatment status was correctly determined in 67.7 per cent (that is either true positive or true negative). Conversely, pathologists considered 44.7 per cent of their assessments as difficult/uncertain. Of these, the treatment status was correctly determined in 56.2 per cent. *Figure 1b* details how ratings of certainty and difficulty related to correct assessment. *Figure 2* shows images of slides from some of the best and worst scored patients, and the overlap of histomorphological features between treatment-naive and treated patients.

Histological features that were most frequently stated to allow distinction between treated and treatment-naive patients were: reduced cancer cell density (10 of 18 pathologists), presence of mucin pools (10 of 18), cell degeneration (9 of 18), and fibro(myxoid) stromal changes (8 of 18) (*Table 4*). *Table S2* contains the verbatim free-text answers provided by all 18 pathologists.

**Table 4:** Histological features in pancreatic cancer resections that pathologists identified as indicative of treatment effect

	No. of pathologists
**Stromal changes**	
Mucin pools	10 of 18
Fibro(myxoid) changes	8 of 18
Foamy cells	3 of 18
Histiocytes	1 of 18
**Cellular changes**	
Cellular degenerative changes	9 of 18
Cytoplasmic vacuolization	4 of 18
Nuclear atypia	5 of 18
Clear cell change	4 of 18
**General changes**	
Reduced cancer cell density	10 of 18
Vascular changes	5 of 18
Necrosis	2 of 18
**Others**	
Duodenal protection from treatment effect	2 of 18
Islet hyperplasia	1 of 18
Neural hyperplasia	1 of 18
Tumour insular complexes	1 of 18
Duct destruction	1 of 18
Parenchymal congestion	1 of 18

### Interobserver agreement on the degree of treatment response (part 2)

**Fig. 3:**
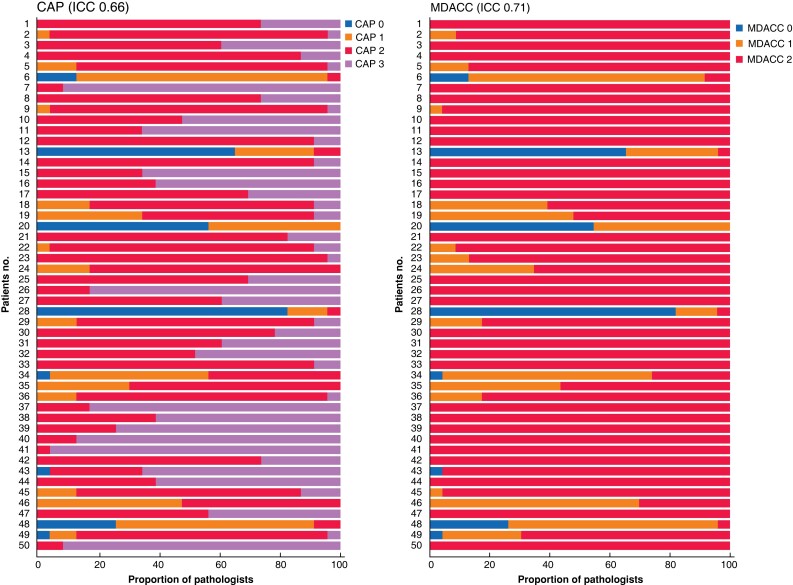
Interobserver agreement among 23 pathologists for each patient Assessments for none of the 50 patients reached 100 per cent consensus among 23 pathologists using the College of American Pathologists (CAP) system. In contrast, 29 of 50 reached 100 per cent consensus among 23 pathologists using the MD Anderson Cancer Center (MDACC)system. Among the 29 patients with 100 per cent consensus using the MDACC system, the intraclass correlation coefficient (ICC) using the CAP system was 0.28. Patients 1–33 were treated with FOLFIRINOX (combination treatment consisting of leucovorin, fluorouracil, irinotecan, and oxaliplatin-based therapy), and patients 34–50 with gemcitabine-based therapy.

Overall, 23 pathologists from 9 countries participated. Interobserver agreement for the CAP system had an ICC of 0.66 (95 per cent c.i. 0.57 to 0.75). For the MDACC system, the ICC was 0.71 (0.63 to 0.79). *Figure 3* shows stacked bar charts for the 50 patients scored. *Tables S3 and S4* detail the scores for each patient.

Using the CAP system, 100 per cent consensus among the participating pathologists was never reached, whereas 100 per cent consensus was reached for 29 of 50 patients with the MDACC system. Among the 29 patients with 100 per cent consensus using the MDACC system, the ICC for the CAP system was 0.28 (0.18 to 0.43). For the remaining 21 patients, the MDACC system reached an ICC of 0.61 (0.47 to 0.77), and the CAP system reached an ICC of 0.62 (0.48 to 0.78).

Eight patients were scored as showing a complete response by at least one pathologist, but 100 per cent consensus was reached in none of these. Among these, three patients (nos 13, 20, and 28) were scored as having a complete response by most pathologists (56.5, 65.2, and 82.6 per cent respectively). Of the remaining scores, on average, 28.3 per cent were CAP 1 or MDACC 1, and 3.6 per cent were CAP 2/3 or MDACC 2. Another five patients (nos 6, 34, 43, 48, and 49) were scored as showing a complete response by only a minority of pathologists (13.0, 4.4, 4.4, 26.1, and 4.4 per cent respectively). On average, among these patients, 45.2 per cent of the remaining scores were CAP 1 or MDACC 1, and 44.3 per cent were CAP 2/3 or MDACC 2.

In the regimen-based sensitivity analysis, 33 patients received FOLFIRINOX-based therapy, and 17 gemcitabine-based therapy. For the CAP system, the ICC was 0.68 in the FOLFIRINOX-based group, and 0.62 in the gemcitabine-based group. In the MDACC system, the ICC was 0.77 in the FOLFIRINOX-based group, and 0.56 in the gemcitabine-based group.

## Discussion

This international interobserver study of current TRS systems and the treatment effect of NAT in resected PDAC evaluated by experienced pathologists has demonstrated that histological identification of the effects of NAT is challenging. Furthermore, this study has shown that interobserver agreements for the CAP and MDACC TRS systems are moderate, with ICCs of 0.66 and 0.71 respectively.

Reduced cancer cell density, stromal reaction, and the presence of stromal mucin pools were the changes that pathologists most frequently regarded as evidence of therapy effect. The results indicate that such histological features are most likely non-specific and may also occur in treatment-naive patients. Both the CAP and MDACC TRS systems are based on the identification of viable tumour cells in relation to the tumour bed before treatment. If pathologists cannot recognize NAT-induced changes reliably, the basis for these TRS systems is arguable. The present findings emphasize the need for a more reproducible system to score the effect of NAT, supporting the recently published ISGPP recommendations to determine the presence of residual tumour burden rather than the extent of tumour regression after NAT^6^.

In this study, the interobserver agreement for both the CAP and MDACC TRS systems was only moderate. As shown in *Fig. 3*, interobserver variation was high for a considerable proportion of patients. Most concerning was the significant interobserver disagreement even for patients who, according to some pathologists, showed a complete pathological response (CAP 0 and MDACC 0). Among patients graded as CAP 0/MDACC 0 by at least one pathologist, none had 100 per cent concordance. This disagreement may have been due to difficulty in distinguishing *in situ* neoplasia from invasive carcinoma or because of cancerization of the ducts and loss of non-neoplastic structures that can be used to identify the location of glands in question^17,18^. The disagreement may also have resulted from difficulty in discerning residual cancer focus/foci in the background of markedly altered non-neoplastic tissues. Although not entirely unexpected, there was considerable divergence of opinion for patients scored as CAP 1/MDACC 1 by at least one pathologist. Indeed, the descriptive criteria distinguishing CAP 1 from CAP 2 (*Table 1*) are not precise, and so leave room for divergent interpretation; the exact number of single cells or small groups of cancer cells that maximally may be present for a patient to fall into CAP 1 is unclear. When it comes to CAP 2, the opinion of each pathologist determines the degree of tumour regression that qualifies as ‘evident’. Conversely, it remains unclear how extensive residual cancer should be for categorization as CAP 3. The difficulty in using the CAP grading system was highlighted by the finding that concordant grading was achieved in 29 of 50 patients using the MDACC TRS system, but in none of 50 using the CAP system. It should be noted that, for the categories MDACC 1 and 2, determination of the amount of residual carcinoma and estimation of the treated tumour bed remain challenging, given that fibrosis may occur for reasons other than tumour regression.

A few other studies^16,19–22^ have investigated interobserver agreement for TRS systems in pancreatic cancer (*Table 5*). Although the outcomes, study materials, and methods vary, the present findings are concordant with those of other studies showing that interobserver agreement is suboptimal for both the CAP and MDACC systems. Unlike previous studies, based on 2–8 participating pathologists, the present study included 23 gastrointestinal/pancreatic pathologists from 9 different countries. Because a large number of pathologists graded each patient, the present study provides deeper insight into the scope of interobserver disagreement for the individual TRS systems by showing the patterns of (dis)agreement (*Fig. 3*). Moreover, it differs from previous analyses in that the sampling methodology was more standardized, and only experienced gastrointestinal/pancreatic pathologists undertook the scoring. Finally, this study differs from earlier studies that used Cohen’s κ or Kendall’s concordance coefficient as outcome measures. The ICC was chosen here, as Cohen’s κ measure is only appropriate for two raters^23^ and because the rank-based Kendall’s concordance coefficient is considered inappropriate for interobserver studies with few categories, given the high probability of tied ranks^24^.

**Table 5:** Interobserver agreement studies of tumour response scoring systems for pancreatic cancer

Reference	No. of patients	No. of graders	Sampling	% of patients for whom assessment reached consensus	Interobserver measure outcomes
Kalimuthu *et al.*^19^	14	4	7–20 slides	CAP: 57.1 MDACC: 85.7	CAP: KCC 0.18–0.40 MDACC: KCC 0.00–0.67
Cacciato Insilla *et al*.^20^	29	2	Complete submission of surgical specimen and overall evaluation of all slides	CAP: 72.4 MDACC: 89.7	CAP: KCC 0.64 MDACC: KCC 0.52
Matsuda *et al*.^21^	97	2	All histology sections	CAP: 71.1 MDACC: 94.8	CAP: Cohen’s κ 0.50 MDACC: Cohen’s κ 0.65
Kameyama *et al*.^16^	30	8	Not specified	CAP: 13.3 MDACC: 83.3	CAP: KCC 0.48 MDACC: KCC 0.53
Chou *et al*.^22^	147	2	Two sections of tumour containing the most malignant tissue	CAP: 66* MDACC: 92*	CAP: Cohen’s κ 0.43 MDACC: Cohen’s κ 0.69
Present study	50	23	Three randomly selected slides from tumour bed	CAP: 0 MDACC: 58	CAP: ICC 0.66 MDACC: ICC 0.71

* From review of original data sets (data not presented in original paper). CAP, College of American Pathologists; MDACC, MD Anderson Cancer Center; KCC, Kendall’s coefficient of concordance; ICC, intraclass correlation coefficient.

The findings of this study should be interpreted with a few limitations in mind. First, it cannot be excluded that some of the patients treated with NAT, whose tissue slides were included in part 1, showed no treatment effect. In these instances, it would have been impossible for the pathologists to identify a treatment effect. However, the effect of such a bias is likely to be limited as pathologists tended to overclassify treatment-naive patients as treated. Second, sampling three slides per patient for the interobserver agreement section could have resulted in either optimistic or pessimistic scores. A random number generator was used to select haematoxylin and eosin-stained slides from each patient to minimize sampling bias in this study. This sampling method does not reflect clinical practice, in which a pathologist would use all of the microscopic slides. However, as tumour regression is known to be heterogeneous within a single tumour, it is necessary to assess the degree of tumour regression independently in each individual slide to arrive at an overall semiquantitative assessment of TRS. Hence, the limited number of slides used in this study is not entirely unrepresentative of clinical practice. Besides, evaluation of many more slides was not deemed practically feasible for this study in terms of data storage and time required from the participating pathologists. In addition, the aim was not to investigate the prognostic or clinical value of TRS, but rather to focus on the assessment of interobserver agreement.

In terms of clinical relevance, the present findings have shown that clinical decision-making and comparison of the effectiveness of different NAT regimens based on histopathological TRS remain troublesome. Moreover, it remains unclear how many tiers provide optimal and clinically relevant risk stratification. Indeed, reducing the number of tiers of a classifier will improve interobserver agreement, but may also result in loss of valuable information for the clinical oncologist. To use TRS to guide selection of adjuvant therapy in routine clinical practice, the causes of interobserver variation described in this study need to be addressed. The lack of reproducibility of these TRS systems is also relevant in the clinical research setting, particularly in clinical trials evaluating the efficacy of NAT regimens based on tumour response scores.

Based on the findings from this study, future research should focus where possible on the development of a TRS system that assesses residual tumour burden in pancreatic cancer and employs reproducible criteria that facilitate greater interobserver concordance. This may include artificial intelligence-based segmentation strategies to improve objectivity^25^. During the development of such a TRS system, the variety of neoadjuvant regimens needs to be considered. As the present sensitivity analysis has shown, interobserver variation may be better in the FOLFIRINOX group for both the CAP and MDACC systems. However, the groups in the sensitivity analyses were relatively small and, as such, their results are inconclusive. Finally, in addition to histopathological TRS systems, genomic alterations detected in pretreatment biopsy material, and serum-based and imaging markers have shown promise in recent response scoring studies^8,10,26^, and ought to be explored further. Among these, serum carbohydrate antigen 19-9 and other blood-based biomarkers, CT-radiomics features, and fluorodeoxyglucose PET currently appear to be more robust^10^.

## Supplementary Material

znac350_Supplementary_Data

## Data Availability

The data used to support the findings of this study are accessible in the *supplementary material.*
