# Supplementary material for: Histopathological tumour response scoring in resected pancreatic cancer following neoadjuvant therapy: international interobserver study (ISGPP-1)
Source: Br J Surg. 2022 Nov 4;110(1):67–75. doi: 10.1093/bjs/znac350 (PMC10364538; doi:10.1093/bjs/znac350)
Supplement: znac350_Supplementary_Data [file znac350_supplementary_data.docx]

**SUPPLEMENTARY MATERIAL**

**Table S1:** Identification of treatment-induced changes in 178 slides by 18 pathologists

| Rater | True positive | False positive | True negative | False negative | Sensitivity | Specificity | Positive predictive value | Negative predictive value |
| --- | --- | --- | --- | --- | --- | --- | --- | --- |
| 1 | 48 | 29 | 61 | 40 | 54.5 | 67.8 | 62.3 | 60.4 |
| 2 | 76 | 60 | 30 | 12 | 86.4 | 33.3 | 55.9 | 71.4 |
| 3 | 60 | 50 | 40 | 28 | 68.2 | 44.4 | 54.5 | 58.8 |
| 4 | 58 | 46 | 44 | 30 | 65.9 | 48.9 | 55.8 | 59.5 |
| 5 | 68 | 49 | 41 | 20 | 77.3 | 45.6 | 58.1 | 67.2 |
| 6 | 70 | 32 | 58 | 17 | 79.5 | 64.4 | 68.6 | 77.3 |
| 7 | 69 | 29 | 61 | 19 | 78.4 | 67.8 | 70.4 | 76.3 |
| 8 | 61 | 48 | 42 | 27 | 69.3 | 46.7 | 56.0 | 60.9 |
| 9 | 56 | 39 | 51 | 32 | 63.6 | 56.7 | 58.9 | 61.4 |
| 10 | 85 | 73 | 17 | 3 | 96.6 | 18.9 | 53.8 | 85.0 |
| 11 | 81 | 44 | 46 | 7 | 92.0 | 51.1 | 64.8 | 86.8 |
| 12 | 70 | 56 | 34 | 18 | 79.5 | 37.8 | 55.6 | 65.4 |
| 13 | 86 | 66 | 24 | 2 | 97.7 | 26.7 | 56.6 | 92.3 |
| 14 | 62 | 44 | 46 | 26 | 70.5 | 51.1 | 58.5 | 63.9 |
| 15 | 66 | 35 | 55 | 22 | 75.0 | 61.1 | 65.3 | 71.4 |
| 16 | 61 | 51 | 39 | 27 | 69.3 | 43.3 | 54.5 | 59.1 |
| 17 | 66 | 31 | 59 | 22 | 75.0 | 65.6 | 68.0 | 72.8 |
| 18 | 64 | 44 | 46 | 24 | 72.7 | 51.1 | 59.3 | 65.7 |
| Total: | 1208 | 825 | 795 | 376 | 76.2 | 49.0 | 59.4 | 67.9 |

**Table S2:** Histologic features used to make the distinction between treated and treatment-naïve pancreatic cancer

| Pathologist | Overall, which histologic features helped you make the distinction between treated and treatment naïve cases? |
| --- | --- |
| 1 | Presence of "monster" cells is typical of treated cases.  Types of fibrosis and cell types of treated tumors.  Tumors within duodenal wall usually do not respond to chemotherapy as tumor in the pancreas. |
| 2 | Tumor cells showing clear cell change. Histiocytes and extracellular mucin pools were also useful features. |
| 3 | Mucin around nerves without cells |
| 4 | Clear cytoplasmic change Mucin pools in background of fibrosis Venous wall change Congestion in normal pancreatic parenchyma |
| 5 | Foamy cells, hyaline fibrosis |
| 6 | 1. Myxoid stromal change with scattered/rare very atypical (degenerative) tumor cells or scattered glands with very flattened tumor cells.  2. Large area of myxoid fibrosis with no tumor cells  3. Stroma with elastotic change  4. Acellular mucin  5. nerves surrounded by myxoid/acellular mucosa and inflammation  5. vascular damage in areas with no or scattered tumor cells |
| 7 | Fibrosis without tumor cells extending into the peri pancreatic fat tissue.  Ducts with signs of destruction and cellular changes suggestive of cell damage and mucinous lakes  Extensive acellular sclerosis, different that typical desmoplastic stroma |
| 8 | Stromal quality. Atypical epithelial cell nuclei. Epithelial cell vacuolation. Dispersion of tumor foci. Areas of necrosis or degeneration. Mucin pools. Vascular changes. But the only reliable way is to have a clinical history. |
| 9 | (1) Degeneration of cells (haphazardly arranged, pyknotic nuclei in cells with abundant cleared cytoplasm)  (2) Degeneration of cells (akin to endocrine atypia)  (3) Many acellular mucin pools in non-mucinous tumor  (4) Mostly single large tumor cells in scar  (5) Extremely paucicellular scar (scar>>> tumor cells) |
| 10 | Degenerative changes of cancer cells, mucin pools, alternation of paucicellular, collagen-rich fibrosis and more cellular and mucoid connective tissue |
| 11 | Presence of  -fibrotic or fibromyxoid stroma  -bizarre nuclei such as irregular, hyperchromatic, binucleated or multinucleated  -cytoplasmic vacuolization and eosinophilia  -swollen cytoplasm and nucleus.  Necrosis alone is not helpful |
| 12 | -Isolated extremely pleomorphic/vacuolated cells |
| 13 | Bizarre often single cells with large irregular nuclei and often abundant cytoplasm; myxoid or fibrous stroma overgrowing and entrapping glands rather than glands intermixed and invading with desmoplastic stroma; large zones of stroma/fibrosis separating areas of viable tumor; "protection" from treatment effect by muscularis propria of duodenum; uncommonly, large foamy cells with very tiny pyknotic nuclei |
| 14 | Cytoplasmic clearing. |
| 15 | Features of treatment effect:  1. Scant/ reduced tumor cellularity compared to stroma.  2. Fibrosis in adjacent parenchyma, blurring tumor-normal interface.  3. Fibrosis encompassing adjacent vessels.  4. Thick-walled vessels with intimal fibrosis, neural hyperplasia, and islet hyperplasia in adjacent stroma.  5. Confluent necrosis.  6. Acellular mucin pools.  7. Cytopathic changes in tumor cells, particularly foamy cell change.  8. Prominent tumor-insular complexes may be seen. |
| 16 | Stroma modifications  Regressive changes in neoplastic cells  Vascular changes  Dispersed areas of neoplastic cells |
| 17 | -Fibrotic, expansile scar  -Foamy macrophages and acellular mucin  -Clear, vacuolated cytoplasm with pyknotic nuclei |
| 18 | The combination (of three factors) of dense hyalinization with sparsity of cells that also show marked degenerative cytology is essentially the only picture that made me conclude most likely treatment with some certainty. |

**Table S3:** CAP scores in 50 patients by 23 pathologists

| Rater | 1 | 2 | 3 | 4 | 5 | 6 | 7 | 8 | 9 | 10 | 11 | 12 | 13 | 14 | 15 | 16 | 17 | 18 | 19 | 20 | 21 | 22 | 23 |
| --- | --- | --- | --- | --- | --- | --- | --- | --- | --- | --- | --- | --- | --- | --- | --- | --- | --- | --- | --- | --- | --- | --- | --- |
| Patient |  |  |  |  |  |  |  |  |  |  |  |  |  |  |  |  |  |  |  |  |  |  |  |
| 1 | 2 | 2 | 3 | 2 | 2 | 2 | 3 | 3 | 2 | 3 | 2 | 3 | 2 | 2 | 2 | 2 | 3 | 2 | 2 | 2 | 2 | 2 | 2 |
| 2 | 2 | 1 | 2 | 2 | 2 | 2 | 2 | 2 | 2 | 3 | 2 | 2 | 2 | 2 | 2 | 2 | 2 | 2 | 2 | 2 | 2 | 2 | 2 |
| 3 | 2 | 2 | 2 | 2 | 3 | 3 | 2 | 2 | 3 | 3 | 2 | 2 | 3 | 2 | 2 | 2 | 3 | 2 | 2 | 3 | 3 | 3 | 2 |
| 4 | 2 | 2 | 3 | 2 | 2 | 2 | 3 | 2 | 2 | 3 | 2 | 2 | 2 | 2 | 2 | 2 | 2 | 2 | 2 | 2 | 2 | 2 | 2 |
| 5 | 2 | 1 | 2 | 2 | 2 | 3 | 2 | 2 | 2 | 2 | 2 | 1 | 2 | 2 | 2 | 2 | 1 | 2 | 2 | 2 | 2 | 2 | 2 |
| 6 | 1 | 1 | 0 | 1 | 1 | 1 | 1 | 0 | 2 | 0 | 1 | 1 | 1 | 1 | 1 | 1 | 1 | 1 | 1 | 1 | 1 | 1 | 1 |
| 7 | 3 | 3 | 3 | 3 | 3 | 3 | 3 | 3 | 3 | 3 | 3 | 2 | 3 | 3 | 3 | 3 | 3 | 3 | 3 | 3 | 3 | 3 | 2 |
| 8 | 2 | 2 | 3 | 2 | 2 | 2 | 3 | 2 | 2 | 3 | 2 | 2 | 3 | 3 | 2 | 2 | 2 | 3 | 2 | 2 | 2 | 2 | 2 |
| 9 | 2 | 2 | 1 | 2 | 2 | 2 | 3 | 2 | 2 | 2 | 2 | 2 | 2 | 2 | 2 | 2 | 2 | 2 | 2 | 2 | 2 | 2 | 2 |
| 10 | 2 | 3 | 3 | 2 | 2 | 3 | 3 | 3 | 3 | 2 | 2 | 3 | 3 | 2 | 3 | 2 | 3 | 2 | 2 | 3 | 2 | 3 | 2 |
| 11 | 2 | 3 | 3 | 2 | 3 | 3 | 3 | 2 | 3 | 3 | 3 | 3 | 3 | 3 | 3 | 2 | 3 | 2 | 2 | 3 | 3 | 2 | 2 |
| 12 | 2 | 2 | 2 | 2 | 2 | 3 | 2 | 2 | 2 | 2 | 2 | 2 | 2 | 2 | 2 | 2 | 2 | 2 | 2 | 2 | 3 | 2 | 2 |
| 13 | 1 | 2 | 1 | 0 | 0 | 0 | 0 | 0 | 1 | 0 | 0 | 1 | 1 | 0 | 0 | 0 | 0 | 0 | 0 | 0 | 0 | 1 | 2 |
| 14 | 2 | 2 | 2 | 2 | 2 | 2 | 3 | 3 | 2 | 2 | 2 | 2 | 2 | 2 | 2 | 2 | 2 | 2 | 2 | 2 | 2 | 2 | 2 |
| 15 | 2 | 2 | 3 | 3 | 3 | 3 | 2 | 3 | 3 | 3 | 2 | 3 | 3 | 3 | 3 | 3 | 2 | 3 | 2 | 3 | 3 | 2 | 2 |
| 16 | 2 | 3 | 3 | 3 | 3 | 3 | 3 | 3 | 2 | 3 | 2 | 3 | 2 | 3 | 2 | 2 | 3 | 3 | 2 | 2 | 3 | 3 | 2 |
| 17 | 2 | 3 | 3 | 2 | 2 | 3 | 2 | 2 | 2 | 3 | 2 | 3 | 3 | 2 | 2 | 2 | 2 | 3 | 2 | 2 | 2 | 2 | 2 |
| 18 | 1 | 2 | 3 | 2 | 2 | 1 | 2 | 2 | 3 | 2 | 1 | 2 | 2 | 2 | 2 | 2 | 2 | 2 | 2 | 2 | 2 | 1 | 2 |
| 19 | 1 | 2 | 2 | 2 | 1 | 1 | 3 | 1 | 2 | 3 | 1 | 2 | 1 | 2 | 2 | 2 | 1 | 2 | 2 | 2 | 2 | 1 | 2 |
| 20 | 0 | 0 | 0 | 1 | 1 | 0 | 0 | 0 | 1 | 0 | 1 | 0 | 1 | 0 | 0 | 1 | 0 | 1 | 0 | 1 | 1 | 1 | 0 |
| 21 | 2 | 2 | 2 | 2 | 2 | 2 | 3 | 2 | 2 | 2 | 2 | 2 | 3 | 2 | 2 | 2 | 2 | 2 | 2 | 3 | 3 | 2 | 2 |
| 22 | 2 | 2 | 2 | 2 | 2 | 1 | 3 | 2 | 2 | 2 | 2 | 2 | 2 | 2 | 2 | 2 | 2 | 3 | 2 | 2 | 2 | 2 | 2 |
| 23 | 2 | 2 | 3 | 2 | 2 | 2 | 2 | 2 | 2 | 2 | 2 | 2 | 2 | 2 | 2 | 2 | 2 | 2 | 2 | 2 | 2 | 2 | 2 |
| 24 | 1 | 2 | 2 | 2 | 1 | 2 | 2 | 2 | 2 | 2 | 2 | 2 | 2 | 2 | 1 | 2 | 1 | 2 | 2 | 2 | 2 | 2 | 2 |
| 25 | 2 | 3 | 2 | 2 | 2 | 2 | 3 | 2 | 2 | 3 | 2 | 3 | 3 | 2 | 2 | 2 | 2 | 3 | 2 | 2 | 3 | 2 | 2 |
| 26 | 3 | 3 | 3 | 3 | 3 | 3 | 3 | 2 | 3 | 3 | 2 | 3 | 3 | 3 | 3 | 3 | 3 | 3 | 3 | 3 | 3 | 2 | 2 |
| 27 | 2 | 2 | 2 | 2 | 2 | 3 | 3 | 2 | 2 | 3 | 2 | 3 | 3 | 2 | 2 | 2 | 2 | 3 | 3 | 3 | 3 | 2 | 2 |
| 28 | 0 | 0 | 0 | 0 | 0 | 0 | 1 | 0 | 0 | 2 | 0 | 0 | 0 | 0 | 0 | 0 | 1 | 0 | 0 | 0 | 0 | 1 | 0 |
| 29 | 1 | 2 | 3 | 2 | 2 | 2 | 2 | 2 | 2 | 3 | 2 | 2 | 1 | 2 | 1 | 2 | 2 | 2 | 2 | 2 | 2 | 2 | 2 |
| 30 | 2 | 2 | 2 | 2 | 2 | 3 | 2 | 2 | 2 | 2 | 2 | 2 | 3 | 2 | 2 | 2 | 3 | 2 | 2 | 3 | 3 | 2 | 2 |
| 31 | 3 | 2 | 3 | 2 | 2 | 3 | 3 | 2 | 2 | 3 | 2 | 3 | 2 | 2 | 2 | 2 | 2 | 3 | 2 | 3 | 3 | 2 | 2 |
| 32 | 2 | 2 | 3 | 2 | 2 | 2 | 3 | 2 | 2 | 3 | 3 | 3 | 2 | 2 | 3 | 2 | 3 | 3 | 3 | 3 | 3 | 2 | 2 |
| 33 | 2 | 2 | 2 | 2 | 2 | 2 | 2 | 2 | 3 | 2 | 2 | 2 | 2 | 2 | 2 | 2 | 2 | 2 | 2 | 2 | 3 | 2 | 2 |
| 34 | 1 | 1 | 1 | 2 | 2 | 1 | 2 | 1 | 2 | 2 | 1 | 1 | 1 | 1 | 2 | 1 | 1 | 2 | 2 | 0 | 2 | 1 | 2 |
| 35 | 1 | 2 | 1 | 2 | 2 | 2 | 2 | 1 | 2 | 2 | 1 | 2 | 2 | 2 | 2 | 2 | 1 | 2 | 2 | 2 | 2 | 1 | 1 |
| 36 | 2 | 2 | 2 | 2 | 2 | 2 | 2 | 2 | 3 | 2 | 2 | 1 | 2 | 1 | 2 | 2 | 2 | 2 | 2 | 2 | 2 | 2 | 1 |
| 37 | 3 | 3 | 3 | 3 | 3 | 3 | 3 | 3 | 3 | 2 | 2 | 3 | 3 | 2 | 3 | 3 | 3 | 3 | 3 | 3 | 3 | 3 | 2 |
| 38 | 2 | 2 | 3 | 3 | 2 | 3 | 3 | 3 | 3 | 2 | 2 | 3 | 2 | 2 | 3 | 2 | 3 | 3 | 3 | 3 | 3 | 3 | 2 |
| 39 | 3 | 3 | 3 | 2 | 3 | 2 | 3 | 3 | 3 | 3 | 2 | 3 | 3 | 2 | 2 | 3 | 3 | 3 | 3 | 3 | 3 | 3 | 2 |
| 40 | 3 | 3 | 3 | 3 | 3 | 3 | 3 | 3 | 2 | 3 | 3 | 3 | 3 | 3 | 3 | 3 | 3 | 3 | 3 | 3 | 3 | 2 | 2 |
| 41 | 3 | 3 | 3 | 3 | 3 | 3 | 3 | 3 | 3 | 3 | 2 | 3 | 3 | 3 | 3 | 3 | 3 | 3 | 3 | 3 | 3 | 3 | 3 |
| 42 | 2 | 2 | 3 | 2 | 2 | 3 | 3 | 2 | 2 | 2 | 2 | 2 | 2 | 2 | 2 | 2 | 2 | 3 | 3 | 2 | 3 | 2 | 2 |
| 43 | 2 | 3 | 3 | 3 | 3 | 3 | 3 | 2 | 2 | 3 | 2 | 3 | 3 | 2 | 3 | 2 | 3 | 3 | 2 | 3 | 3 | 3 | 0 |
| 44 | 2 | 3 | 3 | 3 | 3 | 3 | 3 | 2 | 2 | 3 | 2 | 3 | 3 | 3 | 3 | 2 | 2 | 3 | 2 | 3 | 3 | 2 | 2 |
| 45 | 1 | 2 | 2 | 2 | 2 | 2 | 3 | 1 | 2 | 2 | 2 | 2 | 1 | 2 | 2 | 2 | 2 | 3 | 2 | 2 | 3 | 2 | 2 |
| 46 | 1 | 1 | 2 | 2 | 1 | 2 | 2 | 1 | 2 | 2 | 1 | 2 | 2 | 1 | 2 | 2 | 1 | 2 | 1 | 1 | 2 | 1 | 1 |
| 47 | 2 | 3 | 3 | 2 | 2 | 3 | 3 | 2 | 3 | 2 | 2 | 3 | 2 | 2 | 3 | 2 | 2 | 3 | 2 | 3 | 3 | 2 | 2 |
| 48 | 1 | 0 | 1 | 1 | 1 | 1 | 2 | 0 | 2 | 1 | 1 | 1 | 1 | 0 | 1 | 0 | 1 | 1 | 0 | 1 | 1 | 1 | 0 |
| 49 | 2 | 2 | 0 | 2 | 2 | 2 | 3 | 2 | 2 | 2 | 2 | 2 | 2 | 2 | 2 | 2 | 1 | 2 | 2 | 2 | 2 | 1 | 2 |
| 50 | 3 | 3 | 3 | 3 | 3 | 3 | 3 | 3 | 3 | 3 | 3 | 3 | 3 | 2 | 3 | 3 | 3 | 3 | 3 | 3 | 3 | 3 | 2 |

**Table S4:** MDACC scores in 50 patients by 23 pathologists

| Rater | 1 | 2 | 3 | 4 | 5 | 6 | 7 | 8 | 9 | 10 | 11 | 12 | 13 | 14 | 15 | 16 | 17 | 18 | 19 | 20 | 21 | 22 | 23 |
| --- | --- | --- | --- | --- | --- | --- | --- | --- | --- | --- | --- | --- | --- | --- | --- | --- | --- | --- | --- | --- | --- | --- | --- |
| Patient |  |  |  |  |  |  |  |  |  |  |  |  |  |  |  |  |  |  |  |  |  |  |  |
| 1 | 2 | 2 | 2 | 2 | 2 | 2 | 2 | 2 | 2 | 2 | 2 | 2 | 2 | 2 | 2 | 2 | 2 | 2 | 2 | 2 | 2 | 2 | 2 |
| 2 | 2 | 1 | 2 | 2 | 2 | 2 | 2 | 2 | 2 | 2 | 2 | 2 | 2 | 2 | 2 | 2 | 2 | 2 | 2 | 2 | 2 | 2 | 1 |
| 3 | 2 | 2 | 2 | 2 | 2 | 2 | 2 | 2 | 2 | 2 | 2 | 2 | 2 | 2 | 2 | 2 | 2 | 2 | 2 | 2 | 2 | 2 | 2 |
| 4 | 2 | 2 | 2 | 2 | 2 | 2 | 2 | 2 | 2 | 2 | 2 | 2 | 2 | 2 | 2 | 2 | 2 | 2 | 2 | 2 | 2 | 2 | 2 |
| 5 | 2 | 1 | 2 | 2 | 2 | 2 | 2 | 2 | 2 | 2 | 2 | 1 | 2 | 2 | 2 | 2 | 1 | 2 | 2 | 2 | 2 | 2 | 2 |
| 6 | 1 | 1 | 0 | 1 | 1 | 1 | 1 | 0 | 2 | 0 | 1 | 1 | 1 | 1 | 1 | 1 | 1 | 1 | 2 | 1 | 1 | 1 | 1 |
| 7 | 2 | 2 | 2 | 2 | 2 | 2 | 2 | 2 | 2 | 2 | 2 | 2 | 2 | 2 | 2 | 2 | 2 | 2 | 2 | 2 | 2 | 2 | 2 |
| 8 | 2 | 2 | 2 | 2 | 2 | 2 | 2 | 2 | 2 | 2 | 2 | 2 | 2 | 2 | 2 | 2 | 2 | 2 | 2 | 2 | 2 | 2 | 2 |
| 9 | 2 | 2 | 1 | 2 | 2 | 2 | 2 | 2 | 2 | 2 | 2 | 2 | 2 | 2 | 2 | 2 | 2 | 2 | 2 | 2 | 2 | 2 | 2 |
| 10 | 2 | 2 | 2 | 2 | 2 | 2 | 2 | 2 | 2 | 2 | 2 | 2 | 2 | 2 | 2 | 2 | 2 | 2 | 2 | 2 | 2 | 2 | 2 |
| 11 | 2 | 2 | 2 | 2 | 2 | 2 | 2 | 2 | 2 | 2 | 2 | 2 | 2 | 2 | 2 | 2 | 2 | 2 | 2 | 2 | 2 | 2 | 2 |
| 12 | 2 | 2 | 2 | 2 | 2 | 2 | 2 | 2 | 2 | 2 | 2 | 2 | 2 | 2 | 2 | 2 | 2 | 2 | 2 | 2 | 2 | 2 | 2 |
| 13 | 1 | 1 | 1 | 0 | 0 | 0 | 0 | 0 | 1 | 0 | 0 | 1 | 1 | 0 | 0 | 0 | 0 | 0 | 0 | 0 | 0 | 1 | 2 |
| 14 | 2 | 2 | 2 | 2 | 2 | 2 | 2 | 2 | 2 | 2 | 2 | 2 | 2 | 2 | 2 | 2 | 2 | 2 | 2 | 2 | 2 | 2 | 2 |
| 15 | 2 | 2 | 2 | 2 | 2 | 2 | 2 | 2 | 2 | 2 | 2 | 2 | 2 | 2 | 2 | 2 | 2 | 2 | 2 | 2 | 2 | 2 | 2 |
| 16 | 2 | 2 | 2 | 2 | 2 | 2 | 2 | 2 | 2 | 2 | 2 | 2 | 2 | 2 | 2 | 2 | 2 | 2 | 2 | 2 | 2 | 2 | 2 |
| 17 | 2 | 2 | 2 | 2 | 2 | 2 | 2 | 2 | 2 | 2 | 2 | 2 | 2 | 2 | 2 | 2 | 2 | 2 | 2 | 2 | 2 | 2 | 2 |
| 18 | 1 | 2 | 2 | 2 | 1 | 1 | 2 | 1 | 2 | 2 | 1 | 2 | 2 | 2 | 2 | 1 | 2 | 2 | 2 | 1 | 2 | 1 | 1 |
| 19 | 1 | 2 | 2 | 2 | 1 | 1 | 2 | 1 | 2 | 2 | 1 | 2 | 1 | 2 | 2 | 1 | 1 | 1 | 2 | 2 | 2 | 1 | 1 |
| 20 | 0 | 0 | 0 | 1 | 1 | 0 | 0 | 0 | 1 | 0 | 1 | 0 | 1 | 0 | 0 | 1 | 0 | 1 | 0 | 1 | 1 | 1 | 0 |
| 21 | 2 | 2 | 2 | 2 | 2 | 2 | 2 | 2 | 2 | 2 | 2 | 2 | 2 | 2 | 2 | 2 | 2 | 2 | 2 | 2 | 2 | 2 | 2 |
| 22 | 2 | 2 | 2 | 2 | 2 | 1 | 2 | 1 | 2 | 2 | 2 | 2 | 2 | 2 | 2 | 2 | 2 | 2 | 2 | 2 | 2 | 2 | 2 |
| 23 | 2 | 2 | 2 | 2 | 2 | 2 | 2 | 1 | 2 | 2 | 2 | 1 | 2 | 2 | 2 | 1 | 2 | 2 | 2 | 2 | 2 | 2 | 2 |
| 24 | 1 | 2 | 2 | 2 | 1 | 2 | 2 | 1 | 2 | 2 | 2 | 1 | 2 | 2 | 1 | 1 | 1 | 1 | 2 | 2 | 2 | 2 | 2 |
| 25 | 2 | 2 | 2 | 2 | 2 | 2 | 2 | 2 | 2 | 2 | 2 | 2 | 2 | 2 | 2 | 2 | 2 | 2 | 2 | 2 | 2 | 2 | 2 |
| 26 | 2 | 2 | 2 | 2 | 2 | 2 | 2 | 2 | 2 | 2 | 2 | 2 | 2 | 2 | 2 | 2 | 2 | 2 | 2 | 2 | 2 | 2 | 2 |
| 27 | 2 | 2 | 2 | 2 | 2 | 2 | 2 | 2 | 2 | 2 | 2 | 2 | 2 | 2 | 2 | 2 | 2 | 2 | 2 | 2 | 2 | 2 | 2 |
| 28 | 0 | 0 | 0 | 0 | 0 | 0 | 1 | 0 | 0 | 2 | 0 | 0 | 0 | 0 | 0 | 0 | 1 | 0 | 0 | 0 | 0 | 1 | 0 |
| 29 | 1 | 2 | 2 | 2 | 2 | 1 | 2 | 2 | 2 | 2 | 2 | 2 | 1 | 2 | 1 | 2 | 2 | 2 | 2 | 2 | 2 | 2 | 2 |
| 30 | 2 | 2 | 2 | 2 | 2 | 2 | 2 | 2 | 2 | 2 | 2 | 2 | 2 | 2 | 2 | 2 | 2 | 2 | 2 | 2 | 2 | 2 | 2 |
| 31 | 2 | 2 | 2 | 2 | 2 | 2 | 2 | 2 | 2 | 2 | 2 | 2 | 2 | 2 | 2 | 2 | 2 | 2 | 2 | 2 | 2 | 2 | 2 |
| 32 | 2 | 2 | 2 | 2 | 2 | 2 | 2 | 2 | 2 | 2 | 2 | 2 | 2 | 2 | 2 | 2 | 2 | 2 | 2 | 2 | 2 | 2 | 2 |
| 33 | 2 | 2 | 2 | 2 | 2 | 2 | 2 | 2 | 2 | 2 | 2 | 2 | 2 | 2 | 2 | 2 | 2 | 2 | 2 | 2 | 2 | 2 | 2 |
| 34 | 1 | 1 | 1 | 2 | 2 | 1 | 1 | 1 | 1 | 2 | 1 | 1 | 1 | 1 | 1 | 1 | 1 | 2 | 2 | 0 | 2 | 1 | 1 |
| 35 | 1 | 2 | 1 | 2 | 2 | 1 | 2 | 1 | 2 | 2 | 1 | 2 | 2 | 2 | 2 | 1 | 1 | 2 | 2 | 1 | 2 | 1 | 1 |
| 36 | 2 | 2 | 2 | 2 | 2 | 1 | 2 | 2 | 2 | 2 | 2 | 1 | 2 | 1 | 2 | 2 | 2 | 2 | 2 | 2 | 2 | 2 | 1 |
| 37 | 2 | 2 | 2 | 2 | 2 | 2 | 2 | 2 | 2 | 2 | 2 | 2 | 2 | 2 | 2 | 2 | 2 | 2 | 2 | 2 | 2 | 2 | 2 |
| 38 | 2 | 2 | 2 | 2 | 2 | 2 | 2 | 2 | 2 | 2 | 2 | 2 | 2 | 2 | 2 | 2 | 2 | 2 | 2 | 2 | 2 | 2 | 2 |
| 39 | 2 | 2 | 2 | 2 | 2 | 2 | 2 | 2 | 2 | 2 | 2 | 2 | 2 | 2 | 2 | 2 | 2 | 2 | 2 | 2 | 2 | 2 | 2 |
| 40 | 2 | 2 | 2 | 2 | 2 | 2 | 2 | 2 | 2 | 2 | 2 | 2 | 2 | 2 | 2 | 2 | 2 | 2 | 2 | 2 | 2 | 2 | 2 |
| 41 | 2 | 2 | 2 | 2 | 2 | 2 | 2 | 2 | 2 | 2 | 2 | 2 | 2 | 2 | 2 | 2 | 2 | 2 | 2 | 2 | 2 | 2 | 2 |
| 42 | 2 | 2 | 2 | 2 | 2 | 2 | 2 | 2 | 2 | 2 | 2 | 2 | 2 | 2 | 2 | 2 | 2 | 2 | 2 | 2 | 2 | 2 | 2 |
| 43 | 2 | 2 | 2 | 2 | 2 | 2 | 2 | 2 | 2 | 2 | 2 | 2 | 2 | 2 | 2 | 2 | 2 | 2 | 2 | 2 | 2 | 2 | 0 |
| 44 | 2 | 2 | 2 | 2 | 2 | 2 | 2 | 2 | 2 | 2 | 2 | 2 | 2 | 2 | 2 | 2 | 2 | 2 | 2 | 2 | 2 | 2 | 2 |
| 45 | 2 | 2 | 2 | 2 | 2 | 2 | 2 | 1 | 2 | 2 | 2 | 2 | 2 | 2 | 2 | 2 | 2 | 2 | 2 | 2 | 2 | 2 | 2 |
| 46 | 1 | 1 | 2 | 2 | 1 | 2 | 1 | 1 | 2 | 2 | 1 | 1 | 1 | 1 | 1 | 1 | 1 | 2 | 1 | 1 | 2 | 1 | 1 |
| 47 | 2 | 2 | 2 | 2 | 2 | 2 | 2 | 2 | 2 | 2 | 2 | 2 | 2 | 2 | 2 | 2 | 2 | 2 | 2 | 2 | 2 | 2 | 2 |
| 48 | 1 | 0 | 1 | 1 | 1 | 1 | 1 | 0 | 2 | 1 | 1 | 1 | 1 | 0 | 1 | 0 | 1 | 1 | 0 | 1 | 1 | 1 | 0 |
| 49 | 2 | 2 | 0 | 2 | 2 | 2 | 2 | 1 | 2 | 2 | 2 | 1 | 2 | 2 | 2 | 2 | 1 | 2 | 2 | 1 | 2 | 1 | 1 |
| 50 | 2 | 2 | 2 | 2 | 2 | 2 | 2 | 2 | 2 | 2 | 2 | 2 | 2 | 2 | 2 | 2 | 2 | 2 | 2 | 2 | 2 | 2 | 2 |
